# XceptRf-Net: A Novel Deep Learning and Machine Learning Approach for Pneumonia Diagnosis

**DOI:** 10.2174/0115734056409581251202221437

**Published:** 2026-03-03

**Authors:** Muhammad Usama Tanveer, Kashif Munir, Syed Ali Jafar Zaidi, Syed Rizwan Hassan, Ateeq Ur Rehman, Salil Bharany

**Affiliations:** 1 Institute of Information Technology, Khwaja Fareed University of Engineering and Information Technology, Rahim Yar Khan 64200, Pakistan; 2 Department of Computer Engineering, Gachon University, Seongnam-si 13120, South Korea; 3 Applied Science Research Center, Applied Science Private University, Amman, Jordan; 4 Computer Science and Engineering, Saveetha School of Engineering, Saveetha Institute of Medical and Technical Sciences, Chennai, Tamil Nadu, India; 5 University Center for Research and Development, Chandigarh University, Mohali, Punjab 140413, India; 6 Chitkara University Institute of Engineering and Technology, Chitkara University, Rajpura, Punjab, India

**Keywords:** Pneumonia diagnosis, Chest X-Rays, XceptRF-Net, Deep learning, Machine learning, COVID-19

## Abstract

**Introduction:**

The objective of this study is to develop an advanced and interpretable diagnostic framework combining deep learning and machine learning for the initial diagnosis of pneumonia with high accuracy in pediatric patients to overcome the critical limitations of existing diagnostic procedures.

**Methods:**

We introduce a hybrid model, XceptRF-Net, that integrates deep feature learning of the Xception convolutional neural network and the probabilistic modelling power of the Random Forest for the classification of Fisher’s iris dataset. The first stage of the model considers the high-level spatial features from the chest X-rays, which are extracted using Xception. Followed by that, they are subsequently mapped to a probabilistic feature space using Random Forest, contributing to the feature representation and the classification robustness. The discriminative capability of the engineered features was tested by different machine learning classifiers such as Logistic Regression (LR), K-Nearest Neighbours (KNN), and Multi-Layer Perceptron (MLP). Fine-tuning and k-fold cross-validation were also performed for generalization purposes and to speed up computation.

**Results:**

The proposed XceptRF-Net framework, established from an experimental study on one dataset with 5,863 pediatric CXRs, has been objectively shown to benefit over conventional methods. Logistic Regression achieved the highest diagnostic accuracy of 98%, which is a validation of the spatial and probabilistic feature learning integration.

**Discussion:**

The effectiveness of the XceptRF-Net model highlights the value of combining deep feature extraction with probabilistic modeling to enhance clinical decision-making.

**Conclusion:**

The findings highlight the theoretical superiority of the integration of convolutional deep features with ensemble learning and the generation of probabilistic features for medical image analysis. The proposed method provides a stable and explainable framework for clinical decision support and has high potential for practical use in real-world systems for pediatric pneumonia screening and diagnosis.

## 1 INTRODUCTION

Pneumonia, a serious disease caused by bacteria, fungi, or viruses, presents a major [1] global health challenge. It has consistently claimed numerous lives over the years, significantly affecting communities around the world. Pneumonia is the leading cause of hospitalization in intensive care units [2] and is the top infectious disease responsible for deaths in developing countries. Community-acquired pneumonia (CAP), in particular, is a major illness impacting both hospitalized [3] and non-hospitalized individuals. The development of pneumonia involves fluid accumulation in the lung’s air sacs, leading [4] to a cloudy appearance. Severe cases of pneumonia are linked to excessive inflammation, a weakened immune response [5], and unstable lipid metabolism. CAP is a critical global health issue, responsible for approximately three million deaths annually. Each year, pneumonia affects over two million children under the age of five [6], according to the World Health Organization.

The WHO highlights that 95% of newly diagnosed pneumonia cases occur in resource-limited countries [7], particularly in Southeast Asia and Africa. Older adults are more vulnerable to pneumonia compared to younger individuals, with about one million people aged 65 and older being hospitalized for pneumonia each year in the United States. Research has shown a high prevalence of CAP in metropolitan China [8], with an overall occurrence rate of approximately 7.13 per 1000 person-years, demanding significant attention. Furthermore, 8-20% of hospitalized CAP patients, especially older adults with other health conditions, develop severe cases, often requiring admission to intensive care units [5]. Approximately 25% of these severe cases need advanced treatments such as respiratory tube insertion to lower fatality rates.

Pneumonia remains a leading cause of morbidity and mortality [9] among children worldwide, particularly in low-resource settings where access to skilled radiologists is limited. Accurate and timely diagnosis is critical for improving outcomes [10], but the manual interpretation of paediatric X-rays presents several challenges. Paediatric pneumonia often exhibits subtle radiographic signs that can be difficult to detect [7], leading to diagnostic variability among radiologists. Furthermore, the high volume of cases places additional strain on healthcare systems, increasing the likelihood of diagnostic errors or delays [11]. These challenges highlight the need for automated detection systems that can assist radiologists in identifying pneumonia more efficiently and accurately.

This study enhances pneumonia detection by integrating deep learning and machine learning techniques. We begin by extracting spatial features from chest X-ray images of patients with pneumonia. These features are then transformed into a new probabilistic feature space using a Random Forest algorithm. To evaluate the effectiveness of both the spatial and probabilisticfeatures,weapplyvariousmachinelearning models. This comprehensive approach allows us to compare different feature sets and assess model performances in medical image analysis more effectively. This research proposes a novel deep learning and machine learning-based feature engineering method for effective pneumonia diagnosis. Our findings demonstrate that the proposed methods achieve superior results compared to conventional methods, thus offering promising prospects for pneumonia detection in clinical settings.

### 1.1 Key Contributions

Our main research contribution is as follows:

- An advanced strategy, Novel XceptRF-Net, is presented for successful feature engineering of pneumonia images. The XceptRF-Net initially collects spatial features from visual input. The RF classifier’s features are then used to produce a new feature set.
- We created a pair of sophisticated deep learning models and three machine learning models by utilizing data from pneumonia images along with extra factors. The performance of the technique is assessed using K-fold cross-validation. Furthermore, we assessed the computational complexity of each method.

The remaining structure of this investigation is detailed below: Section II examines the diagnosis of pneumonia, whereas Section III explains the recommended technique. Section IV explains the experimental outcomes. Section V is the study’s conclusion.

### 1.2 Literature Analysis

This section explores many strategies and methodologies that can be utilized to anticipate the occurrence of Pneumonia in the literature. Table **1** shows past work.

### 1.3 Role of Deep Learning in Pneumonia Diagnosis

This work presents a unique way to diagnose lung illnesses [12], notably COVID-19 and pneumonia, using chest X-ray images. The suggested framework, which uses a combination of soft computing, machine learning, and deep learning techniques, differs from previous COVID-19 detection methods in that it focuses entirely on lung-specific variables. To improve classification accuracy, this approach starts with image augmentation, then extracts and normalizes features based on region-of-interest (ROI). The work offers F-RNN-LSTM, a revolutionary deep learning model that optimizes performance while minimizing computing complexity. The study compares the effectiveness of various machine learning approaches. The proposed F-RNN-LSTM model excels in detection accuracy, precision, recall, F1-score, and specificity, with an impressive 92% score. This study advances the field of lung disease detection by introducing a simplified and efficient technique with promising clinical potential.

The study used a unique machine learning (ML) model intended for automatic detection of distinct kinds of pneumonia [13], which performed better than conventional approaches. The study is divided into phases, with the first focusing on Contrast-Limited Adaptive Histogram Equalization improves image quality. Following the preprocessing step, a hybrid CNN-PCA feature extractor model is used to extract the most significant features from the photos. Following that, a unique Extreme Learning Machine (ELM) model is suggested to identify various pneumonia kinds using the retrieved attributes. The examination of the model indicates its superiority over other approaches in the field, with particularly high accuracy, precision, and recall rates. The binary class classification attained accuracy, precision, and recall scores of 99.83%, 1.0, and 1.0, respectively, whereas the multiclass classification achieved 96.32%, 0.99, and 0.98. These findings highlight the effectiveness of the CLAHE-ELM-CNNPCA model in allowing accurate and speedy pneumonia diagnosis, providing crucial support to medical practitioners in clinical decision-making processes.

In this study, the development of deep learning [14] has helped in the diagnosis of TB. Hence, through the incorporation of deep learning models in the diagnosis of pneumonia, healthcare professionals are in a position to make better treatment plans for patients with pneumonia. A recent study has used six deep learning models: CNN, InceptionResNetV2, Xception, VGG16, ResNet50, and EfficientNetV2L using Adam optimizer and the dataset consisting of 5,856 CXR images. The proposed model achieved a 94% accuracy score. These results highlight the idea that the utilization of deep learning enables significant assistance in the clinical decision-making process that can enhance patient outcomes in pneumonia diagnosis and management.

The study uses conventional chest radiographs [15], and those processed with a threshold filter to conduct preliminary experimental evaluations with existing DLS models such as AlexNet and ResNet50 in conjunction with a SoftMax classifier. The findings indicate that VGG19 surpasses other methods in terms of classification accuracy, achieving 86.97%. A customised VGG19 network is used to merge manually extracted features generated using Continuous Wavelet Transform, Discrete Wavelet Transform, and Grey Level Co-occurrence Matrix with Deep Features gained through Transfer Learning, with an ensemble feature scheme. The performance tests of the customised VGG19, using different classifiers, show that the VGG16 with an RF classifier achieves the greatest accuracy of 95.70.

In areas where children are more likely to contract pneumonia [16], early detection and treatment are critical to lowering mortality rates. This study aims to address this urgent requirement by presenting CNN models for identifying pneumonia using X-ray images. The study looks into the usefulness of various CNN designs by changing and adjusting hyperparameters, and increasing the number of convolutional layers can enhance the performance of the model. A total of six models are evaluated, including two custom models with two and three convolutional layers, along with four pre-trained models: VGG16, VGG19, ResNet50, and Inception-v3. The custom models obtain a validation accuracy of 85.26%. These findings demonstrate the potential of CNN-based approaches for diagnosing pneumonia in X-ray images, with model architectural variables influencing performance outcomes. Further research could explore refining model parameters and researching different architectures to promote accuracy and resilience in pneumonia detection, therefore contributing to better healthcare outcomes for impacted populations.

This research addresses this issue by introducing a novel deep neural network architecture that uses dropout in the convolutional layers to enhance performance [17]. The suggested methodology was trained and evaluated using a dataset consisting of 5856 labelled photos obtained from a medical imaging challenge hosted on Kaggle. The dataset consisted of anterior-posterior chest X-ray pictures obtained from paediatric patients aged one to five years. The images were obtained from Guangzhou Women and Children’s Medical Centre in China. The recommended network demonstrated exceptional performance with accuracy, recall, and precision rates of 97.2%, 97.3%, and 97.4% respectively. The results achieved in this Kaggle competition would have secured the top position, highlighting the competitiveness of the proposed solution compared to the most advanced algorithms currently available for identifying pneumonia using medical scan data.

In response to the critical need for rapid pneumonia diagnosis [18], researchers, specialists, and businesses worldwide are aggressively adopting deep training and imaging processing-based systems that have the ability to swiftly analyze hundreds of X-ray (CT) images. Medical image analysis has emerged as a potential research frontier providing useful tools for disease diagnosis and decision-making, notably during outbreaks like MERS and COVID-19. This research examines recent deep convolutional neural network architectures for automatic binary classification of pneumonia images, with an emphasis on fine-tuned versions of well-known models. The results demonstrate that the fine-tuned version of ResNet50 performs exceptionally well, with training and testing accuracies surpassing 96%. These results emphasize the promise of ResNet50 as a dependable tool for clinical decision support in medical imaging applications as well as the usefulness of deep learning approaches in pneumonia diagnosis.

The Vision Transformer framework is used [19], and Chest X-rays are utilized for the detection of pneumonia. Visual Transformers (ViTs) are highly proficient at capturing the overall spatial relationships of objects in a global context. They are also capable of handling images with varying resolutions, which leads to improved accuracy in detecting pneumonia. The objective of the study is to assess the efficacy of this approach compared to state-of-the-art models using a varied CXR dataset. The results demonstrate the outstanding performance of ViT, achieving an accuracy of 97.61%, a sensitivity of 95%, and a specificity of 97%. The ViT-based approach demonstrates the potential for early pneumonia detection in CXRs, with ample room for further progress in this domain. Nevertheless, the constraints of data scarcity and the significance of real-world validation are recognized. Future research directions include improving interpretability, enhancing model robustness, and conducting clinical trials for practical implementation.

### 1.4 Role of Machine Learning and Deep Learning in Pneumonia Diagnosis

The research paper uses single and collective learning methods for pneumonia identification with a dataset of 6087 chest X-ray images [20]. Key research questions

Focused on diagnostic accuracy, the impact of ensemble learning, and the influence of the number of models in ensembles. Results demonstrated high accuracy for both single and ensemble models, with no significant effect of ensemble size on performance. The single model achieved a 93.52% accuracy score, while the ensemble approach achieved a 94.84% accuracy score.

This research describes the use of deep learning algorithms [21] to distinguish chest X-ray images depending on the presence or absence of pneumonia-related changes. The first trials, which used a convolutional neural network model created in Python, yielded promising results. Despite obtaining a high accuracy of around 90%, concerns about overfitting due to dataset size remain. While the model’s accuracy implies potential utility as a decision assistance tool, there is still much space for development.

### 1.5 Overfitting of the Dataset is a Limitation in this Research Work

This study evaluates the literature to improve medical knowledge in areas where radiologists are scarce [22]. To reduce undesirable consequences, such as mortality in remote areas, our study focuses on improving the early detection of pneumonia. There aren’t many studies in the given dataset that focus on pneumonia detection right now. The introduction of algorithms in this field has the potential to improve healthcare delivery. By using support vector machines (SVMs) for classification and convolutional neural networks (CNNs) for feature extraction, our method shows how effective hyperparameter optimization is at improving model performance. Our goal is to create a dominant pre-trained CNN model and classifier through our experiments so that future developments in pneumonia detection algorithms can build upon it. SVM receives an 80% rating. The goal of this study is to spur the creation of better diagnostic instruments for pneumonia diagnosis in the future.

### 1.6 Role of Federated Learning in Pneumonia Diagnosis

To improve the productivity of machine learning (ML) models [23], a significant increase in the volume and variety of datasets is required. However, traditional lab-based datasets have constraints in training ML models for real-time applications in medical environments such as hospitals. To overcome this difficulty, the use of federated learning (FL) in conjunction with deep learning appears to be a potential option. FL protects data privacy while employing deep neural networks to properly discern visual patterns, hence enhancing detection performance. This proposed approach intends to address the limitations associated with relying on lab-based datasets by allowing for the use of real-time data from hospitals and medical institutes. The confidentiality and privacy of data during the transmission of trained models over the Internet are critical. Furthermore, the deployment of such technology may incur additional costs due to resource usage by clients. As a result, resolving these constraints is critical for successfully integrating this technology into real-world medical applications.

### 1.7 Research Gaps

After reviewing previous research, we discovered some gaps.

• Despite the widespread use of conventional techniques for detecting pneumonia, current research reveals a significant gap in the employment of improved methodologies that balance accuracy and computational efficiency. Moreover, the performance scores in advanced diagnostic research remain suboptimal, highlighting the necessity for enhanced approaches that can deliver higher diagnostic accuracy while reducing computational costs.

## 2 PROPOSED METHODOLOGY

**Fig. (1):**
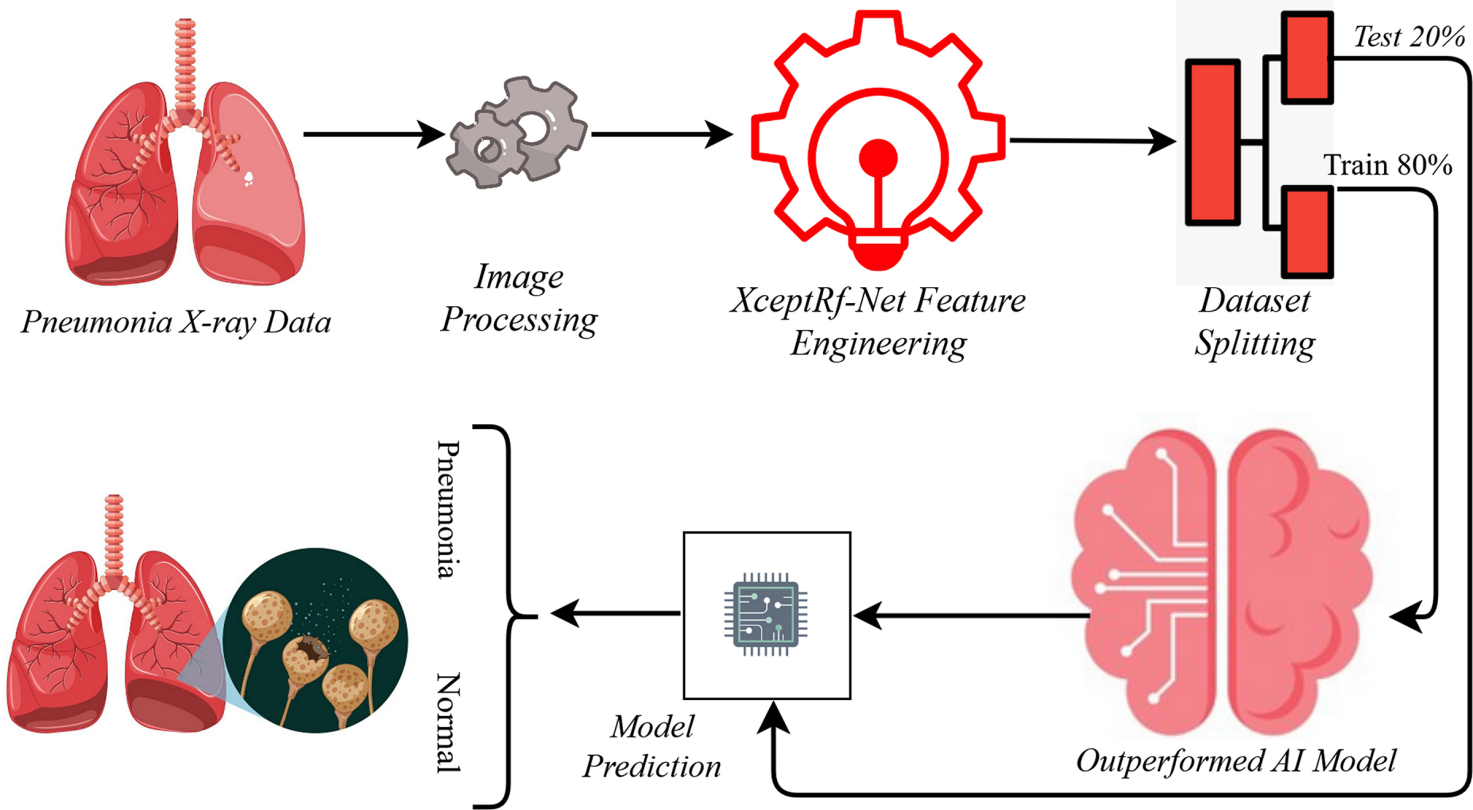
Our innovative approach methodology for the diagnosis of pneumonia.

Figure **1** illustrates our proposed unique methodology for detecting pneumonia. Initially, we obtained standard images for Normal and Pneumonia cases. The image dataset was then processed using basic techniques. The pre-processed images generated a new feature set through transfer learning. These features were divided into training and test sets. The training process utilized 80% of the data to develop machine learning and deep learning models. The trained models were tested on the remaining 20% of the data. The model with the best performance was selected to accurately identify pneumonia.

### 2.1 Dataset

**Fig. (2a-d):**
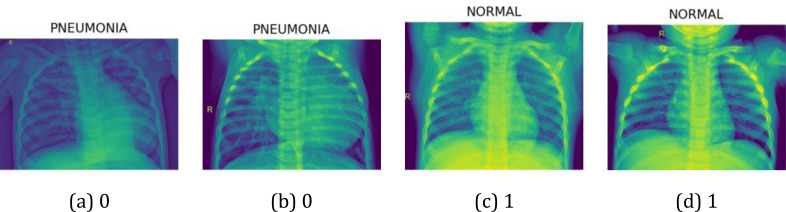
Our novel proposed sample image analysis with the target class.

In this study, we utilized an X-ray image dataset. The dataset includes 5863 paediatric patients aged one to five years, with both anterior and posterior images [24]. Figure **2** describes the target labels for the images. The dataset comprises anterior-posterior chest X-ray images of healthy paediatric patients aged one to five years from the Guangzhou Women and Children’s Medical Center, Guangzhou. All these images were taken while providing clinical care to the participants. To standardize the data, all the radiographs were screened, and only high-quality or plausible and interpretable scans were selected from the examination. Afterwards, each image was reviewed and tagged by two professional diagnosticians. In order to minimize any possible mistakes in grading, a third authoritative view of the evaluation set was employed to provide reliability in training the AI model. The approach we have developed is highly scalable. Our feature extraction process, combined with probabilistic transformation using Random Forest, can be applied to larger datasets with minimal modifications. Additionally, the machine learning models employed are flexible and capable of handling increased data volumes.

### 2.2 Image Processing

To facilitate future study efforts, we performed fundamental image processing tasks. We obtained standard images for Normal and Pneumonia. We refer to the process of acquiring a well-labelled dataset consisting of chest X-ray images from two distinct categories: normal (healthy) lungs and lungs diagnosed with pneumonia. Throughout the process, we standardized the proportions of all imported images. To maintain uniformity in image sizes, we adapted the pixel data of the images into NumPy arrays, which were then transformed into tensors. Following these processes, we assigned numerical values to the images’ target labels, designating ’Pneumonia’ as 0 and ’Normal’ as 1. After completing this phase, we partitioned the dataset into training and testing subsets using an 80:20 split. We then applied Logistic Regression (LR), k-Nearest Neighbours Classifier (KNC), Multi-Layer Perceptron (MLP), and Random Forest (RF) models to the generated feature dataset. Each model was applied to both the raw spatial features and the transformed probabilistic features for comparative analysis.

### 2.3 Novel Proposed Approach XceptRf-Net

**Fig. (3):**
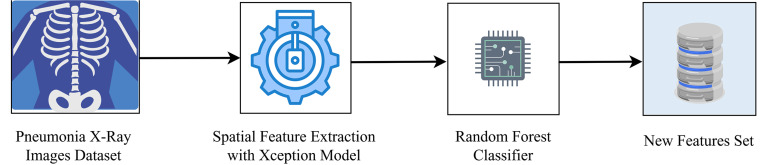
Our novel proposed architectural analysis of the workflow for our innovative feature engineering process for pneumonia diagnosis.

Figure **3** illustrates our proposed architectural analysis. Within this framework, we introduce the XcepRf-Net approach for the feature engineering mechanism, focusing on pediatric pneumonia patient images as depicted in the figure. XcepRf-Net is utilized to identify the optimal feature set. Spatial features are extracted using XcepRf-Net from images of pneumonia-infected patients. Subsequently, a Random Forest is employed to derive probabilistic features. These probabilistic features are then utilized in deploying an advanced machine-learning model for pneumonia diagnosis. The advanced mechanism achieves a high-performance score in predicting the desired outcomes. The CNN is constructed based on the Xception architecture, which has depthwise separable convolutions and is capable of capturing high-level spatial features from chest X-ray images in an efficient way. We specify the main layers of the architecture, such as the input shape (256×256×3), the activation functions (ReLU and sigmoid), and the optimiser settings (Adam with a constant learning rate). Our XceptRF-Net architecture extends these features through a Random Forest classifier, which maps the spatial representations into a probabilistic feature space. This hybrid framework leverages the representational power of deep learning with the ensemble decision-making style of machine learning to construct a robust and interpretable feature engineering pipeline for pneumonia detection.

In the first stage, the input chest X-ray image x is resized to 256×256×3 and fed into the pre-trained model Xception, fine-tuned to the current dataset. The Xception model applies depthwise separable convolutions to capture spatial and semantic-rich representations. We opt to use the feature vectors computed from the final global average pooling layer in Xception, which gives us a high-dimensional representation for each image. Then these extracted spatial features are applied as an input to a Random Forest (RF) classifier, not only for classification, but also to build a new neighbourhood of probabilistic features. In particular, from an individual RF, the class probability distributions for the samples are obtained and are used as additional, informative features to be fed to the next RF. The deep output, after the softmax, is then concatenated with the original spatial features to give a full feature set. This hybrid data representation encodes deep spatial semantics in addition to the decision-level uncertainty, modelled by the ensemble-based RF approach. The last concatenated signal vector is then transmitted to different machine learning classifiers (Logistic Regression, KNN, MLP) for classification performance.

### 2.4 Applied Learning Approaches

We used machine learning and deep learning techniques to implement our innovative approach for the detection of pneumonia. We applied advanced models, including Xception, MLP, KNC, LR, and RF.

### 2.5 Xception Model

The Xception model is highly regarded in the field of deep learning for its outstanding performance in image classification tasks [25]. Its unique design incorporates depthwise separable convolutions, which efficiently capture spatial dependencies in images while significantly reducing computational complexity. In the context of detecting pneumonia from medical images, the Xception model demonstrates impressive effectiveness. By utilizing its deep layers and sophisticated feature extraction capabilities, the model can discern subtle patterns that indicate the presence of pneumonia with a high degree of accuracy. Mathematically, the Xception model can be represented as Eq. **1**:

**Table:** 

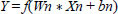	(1)

Here, y represents the output prediction, f denotes the activation function, Wn signifies the weights, Xn represents the input image data, and bn denotes the bias term. The Xception model applies convolutional layers followed by activation functions iteratively to extract hierarchical features from input images. These features are then utilized by subsequent layers to make predictions regarding the presence of pneumonia. Through its sophisticated architecture and mathematical formulation, the Xception model emerges as a powerful tool in the domain of pneumonia detection from medical images. In our usage of the Xception model, there are two motivations: (1) to serve as a deep learning performance baseline through end-to-end classification of images, and (2) to confirm the powerful representations learned by Xception for our follow-up feature engineering process. Despite the fact that the Xception in the XceptRF-Net framework is used for spatial feature extraction, to assess its performance, we have run direct classification experiments with the full Xception and compared it with the performance of traditional machine learning models. This aspect helped us to show that the spatial features learned by Xception are not only classifiable by themselves but also provide a strong ground for further improvement using probabilistic feature transformation using Random Forest.

### 2.6 Multi-Layer Perceptron

The application of Multilayer Perceptron (MLP) models [26] represents a significant advancement in pneumonia detection, owing to their adeptness at discerning intricate patterns from radiographic images. In this innovative approach, the input layer receives intricate pixel data extracted from radiographic images, which are then meticulously processed through one or more hidden layers. Within each hidden layer, numerous neurons engage in intricate computations, wherein they meticulously weigh and integrate their respective inputs. This process is further enriched by the application of an activation function, typically characterized by its nonlinearity, such as the Rectified Linear Unit (ReLU) or Sigmoid function. Mathematically, the intricate computations within each neuron are elegantly encapsulated by Eq. **2**:

**Table:** 

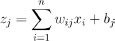	(2)

where zj symbolizes the meticulously weighed sum of inputs for neuron j, wi signifies the weight coefficients connecting input ii to neuron j, xi represents the input values, and bj denotes the neuron’s bias. Ultimately, the output layer of the model synthesizes these meticulously processed features to yield a definitive prediction providing invaluable insights into the likelihood of pneumonia presence.

### 2.7 Random Forest

Random Forest (RF) is a robust and extensively [27] used machine learning technique that excels at classifying tasks like detecting pneumonia in medical image data. RF generates a collection of decision trees, each trained on a random portion of the training data and features. The programme then aggregates the predictions from individual trees to arrive at a final categorization judgement. Let X be the feature matrix representing the input data, Y the target variable indicating the presence or absence of pneumonia, and T the number of decision trees in the forest. Each decision tree (t) is trained recursively to reduce the impurity of the splits produced. The Gini impurity, often known as entropy, is a common impurity metric in radio frequency. For a given input vector x, the projected class label y is determined by a majority vote among all decision trees in the forest Eq. **3**:

**Table:** 

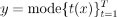	(3)

### 2.8 K Nearest Neighbour Classifier

The K-Nearest Neighbors (KNN) model [28] presents a direct and efficient method for pneumonia detection, utilizing resemblances among medical images. Within this framework, every radiographic image is mapped as a point within a multi-dimensional space, with extracted features aimed at encapsulating pertinent information. Upon presentation of a new image for diagnosis, the KNN algorithm discerns the K nearest neighbors employing a designated distance metric, often Euclidean distance. Subsequently, the labels of these neighboring instances inform the classification of the new image. Mathematically, the KNN algorithm is expressed as follows in Eq. **4**:

**Table:** 

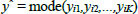	(4)

### 2.9 Logistic Regression

Logistic regression stands as a foundational [29] tool in the realm of pneumonia detection, offering a clear and interpretable framework for classification tasks. In this model, radiographic image features are meticulously examined to ascertain the likelihood of pneumonia presence. Each feature is assigned a weight that reflects its contribution to the classification outcome, and these weights are combined linearly with the feature values. The logistic function, often known as the sigmoid function, is then used in the linear combination to assess the risk of pneumonia. The logistic regression model is mathematically defined as follows in Eq. **5**:

**Table:** 

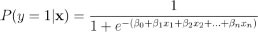	(5)

Where:

• (P(y=1 — x)) represents the probability of pneumonia presence given the input features *x*. *β*_0_*,β*_1_*,...,β_n_* are the model coefficients (weights). *x*_1_*,x*_2_*,...,x_n_* are the input features. *e* is the base of the natural logarithm.

## 3 HYPERPARAMETER TUNING

Table **2** displays the optimal hyperparameters for the machine learning and deep learning models that were deployed. We refined each strategy through k-fold cross-validation, interactive training, and testing. Our research findings demonstrate that by fine-tuning the hyperparameters, we achieved outstanding results for pneumonia diagnosis utilizing medical imaging data.

## 4 RESULTS AND DISCUSSIONS

Our study explores the innovative extraction of features for paediatric pneumonia diagnosis. This section evaluates the effectiveness of the proposed strategy compared to existing approaches through a critical analysis of performance measures.

### 4.1 Experimental Setup

Our experiment uses Google Colab, a cloud-based implementation of Jupyter Notebook. Machine learning approaches are evaluated based on metrics including accuracy, F1 score, precision, and recall. Table **3** provides more information on this.

### 4.2 Xception Model Results

**Fig. (4a-d):**
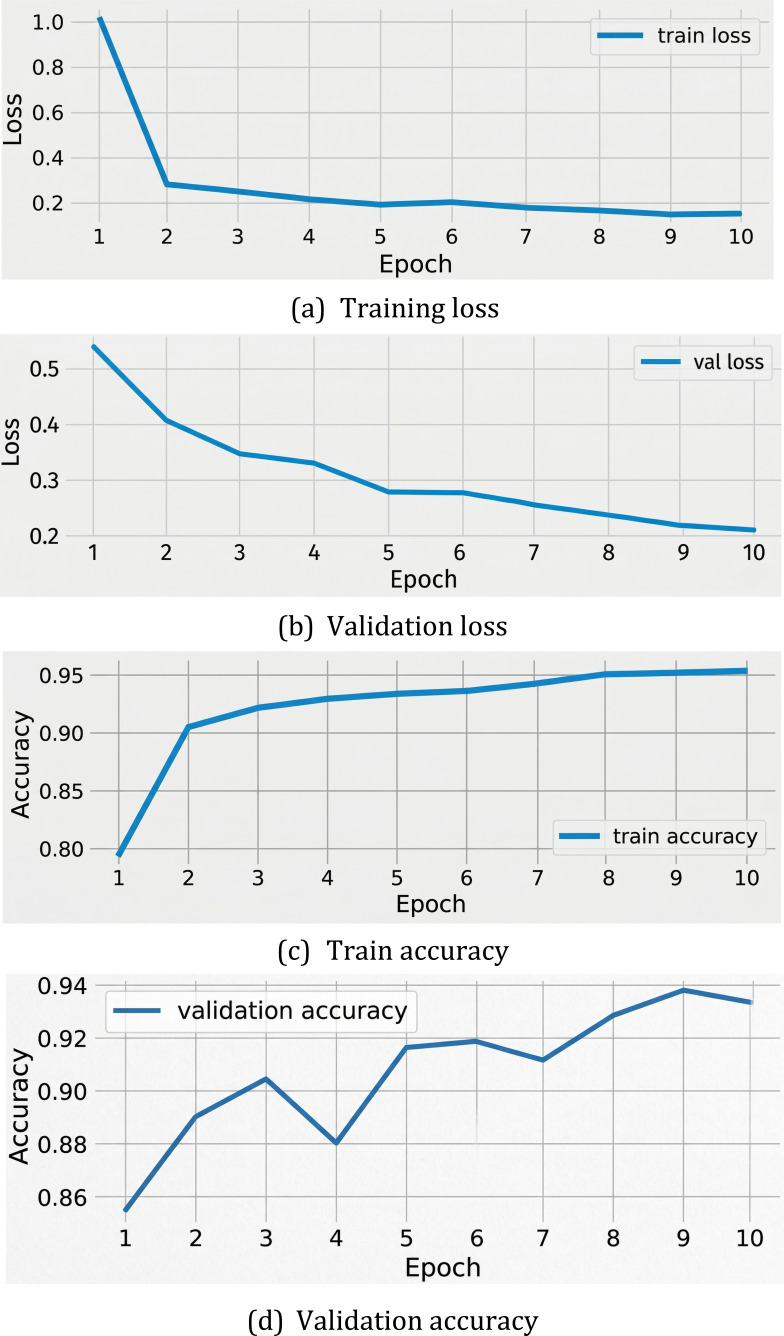
Shows xception model performance analysis.

The Xception model is trained using image data for a total of 20 epochs. The time series analysis indicates notable training loss scores during the initial four epochs of training. Likewise, the validation loss increases during the initial two epochs. Figure **4** displays the results of using the Xception technique to train images. Over time, the Xception model demonstrated enhanced accuracy scores in both training and validation. The accuracy scores for the training and validation sets initially begin at a low level but gradually improve as time progresses. The Xception model’s validation accuracy during training typically varies between 80% and 94%. The correctness of the training data exceeds 90% during the analysis. Table **4** presents an analysis of the performance of the Xception model during testing on previously undisclosed data. According to the statistics, the Xception approach had a diagnostic accuracy rate of 90% for pneumonia. The Xception technique performed well, making it suitable for further research examination.

### 4.3 Performance Analysis using Spatial Feature

Our analysis of the outcome study of Xception indicates that it has the potential to enhance accuracy ratings. The spatial data features extracted from chest images were obtained using Xception and are presented in Table **5**. The sophisticated machine learning techniques employed were trained on these spatial features, and the outcomes are shown below. The results indicate that the RF approach achieved exceptionally high accuracy scores in comparison. However, out of all the methodologies that were assessed, only the KNC method obtained lower scores. This finding suggests a slight increase in the outcomes; however, the performance is still below the expected level for accurately diagnosing the critical condition of pneumonia. Consequently, the use of advanced learning techniques is still required to enhance performance ratings.

### 4.4 Performance Analysis of our Proposed Approach

**Fig. (5):**
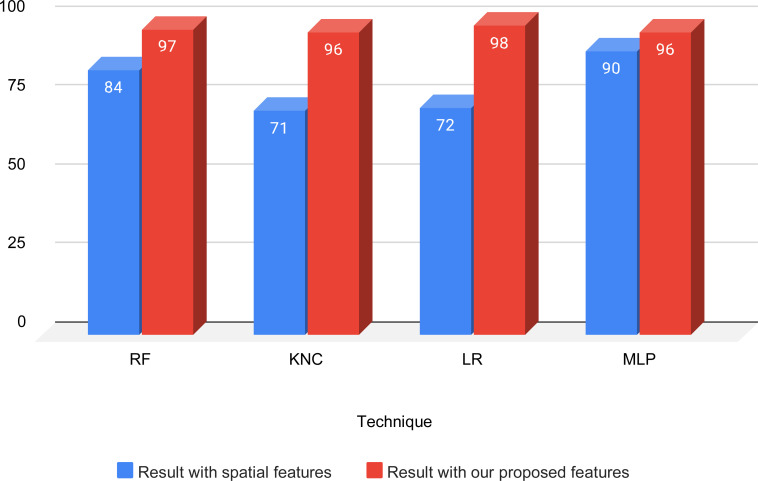
Bar chart-based performance analysis with spatial feature and our proposed newly transferred features.

Table **6** summarizes the performance evaluations of the machine learning models that use the suggested XceptRf-Net features. Interestingly, the results show that the inventive strategy provided in this work improves the performance of the employed methods. All approaches had accuracy values above 96%. Notably, the LR technique outperformed the others with high accuracy ratings of 98%. This asserts that by employing our proposed methodology, the applied methodologies produced outstanding results for diagnosing pneumonia using chest X-ray images. Figure **5** shows a comparison of the performance of classical and newly proposed spatial features using a barcharts. Our research demonstrates that our proposed strategy contributes to great performance outcomes for all methodologies used. The proposed approach provides a more comprehensive view of performance accuracy, as seen in the graph. Our innovative strategy achieved good accuracy scores across all methodologies used in the study. Our results confirm the hypothesis that the incorporation of deep learning and ensemble machine learning leads to improved feature discrimination and model robustness. In particular, the strong performance of our XceptRF-Net framework lends further evidence to the recent trend in the literature that encourages the use of hybrid methodologies that combine spatial representation learning learned through convolutional neural networks with the decision-level probabilistic modelling like Random Forest ensembling. This two-phase learning pipeline not only lifts diagnostic performance from the model, resulting in 98% precision when the logistic regression model is employed, but is also consistent with several feature decorrelation and ensemble diversity theories that are known to mitigate overfitting and to enhance generalisation. Practically, these findings are of significance for the implementation of clinical decision support systems (CDSS). With a more interpretable and reliable diagnosis mechanism provided, our model helps radiologists make faster and more accurate decisions, especially in low-resource settings where expert availability is restricted. Additionally, the probabilistic outputs from the Random Forest module can assist in uncertainty quantification, which is an important aspect for risk-averse applications in pediatric care. Altogether, our work has theoretical implications for the development of hybrid learning structures but also practical implications for AI in the health care domains, supporting the application of such methods in real-world medical diagnoses. To obtain consistent and clear comparison results, 5,863 chest X-ray scan images Table **6** were used were introduced as a common dataset for evaluation, all of which were preprocessed to be rescaled and normalized. During the deep learning-based analysis, these preprocessed images were directly input into an end-to-end fine-tuned Xception model for classification. However, we used high-level spatial features that were extracted from the global average pooling layer of the Xception model, which we used in traditional machine learning experiments. In this study, these features were turned into a probabilistic feature space using the Random Forest algorithm (this formation was integrated into our proposed XceptRF-Net framework). The resulting spatial, statistical, and joint feature vectors were used as input features to classification algorithms, i.e., K-Nearest Neighbors (KNN), Logistic Regression (LR), Random Forest (RF), and Multi-layer Perceptron (MLP). The advantage of this approach is that we were able to compare deep learning raw image-based performance with machine learning feature-based performance in a uniform and representative way.

**Fig. (6):**
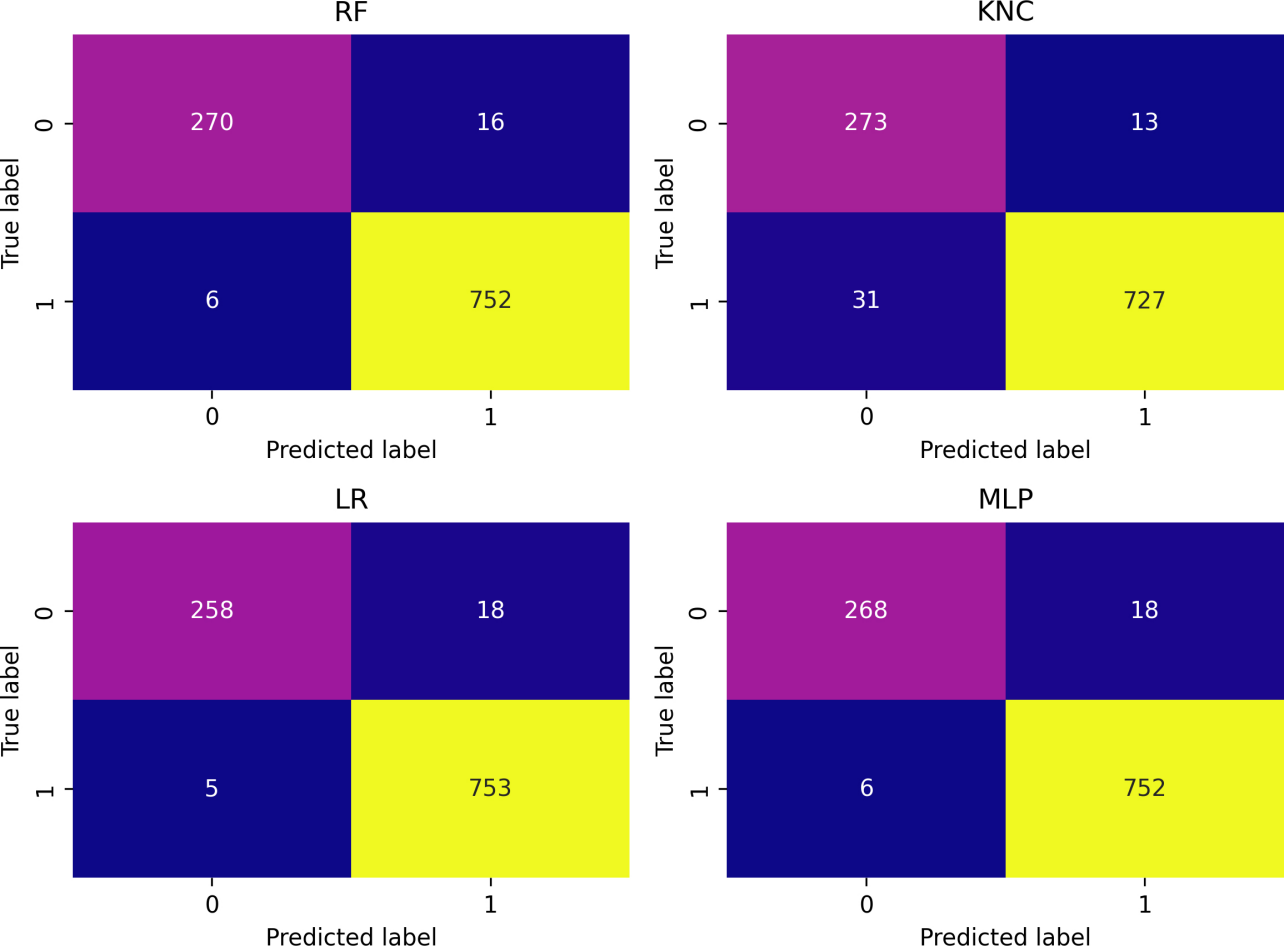
Our novel proposal shows a confusion matrix heatmap graph for pneumonia disease detection.

Figure **6** shows the performance evaluation of the algorithms using the newly proposed features, based on confusion matrix analysis. The analysis shows that all approaches used had lower error rates, reducing misclassifications during the testing phase. The results show that the suggested LR strategy outperforms previous methods with a high correct classification rate and low error rate.

### 4.5 K-fold Validation Analysis

Table **7** shows that we employed the k-fold cross-validation approach, a well-researched strategy for determining whether the machine learning models in use were likely to overfit the data. The validation method was conducted on all five folds in the dataset. Following our recommended methodology, the techniques received perfect marks, demonstrating flawless accuracy with the k-fold procedures. A thorough k-fold validation comparability analysis of different models was conducted. The study found that the strategies regularly produced flawless scores, with the LR model achieving 98% accuracy.

### 4.6 Computational Complexity Analysis

Table **8** summarizes the computational complexity results for the implemented techniques. This analysis suggests that the machine learning methods used achieved lower scores in terms of runtime computational complexity. The MLP technique showed a much higher temporal computational complexity score of 2.8900. In contrast, the proposed LR approach for pneumonia detection using chest X-ray images has a computational complexity of 0.1614 seconds.

### 4.7 State of the Art Studies Comparisons

Table **9** displays a comparative analysis of prior relevant cutting-edge research. The factors being compared are the year, the type of learning utilized, and the proposed method, the accuracy score, the recall score, and the precision score. The analysis indicates that the RF model performed better than the previously used solutions, achieving the highest scores. Our proposed model surpassed the performance of existing studies. The performance improvement is achieved by combining deep spatial features extracted by the Xception-based CNN with the probabilistic mappings provided by the Random Forest algorithm. This leads to a more discriminative and more robust feature space towards noise that often effectively captures both local image patterns and global class distributions. With the high-level representation learning ability of CNN and ensemble decision-making ability of Random Forest incorporated, the model significantly improved robustness and classification accuracy among various manifestations of pneumonia. The architecture of the output layer of the resulting dataset by our proposed XceptRF-Net method: after the Xception model is applied to the chest X-rays, the spatial features are obtained at the global average pooling layer, and a 2048-dimensional feature vector is obtained in each picture. A set of features is then passed to a Random Forest classifier, which produces a probabilistic class distribution that comprises some probability values indicating how confident the prediction is in each class. In our case, as the pneumonia detection is a binary classification problem, an RF yields a 2-dimensional probability feature vector per sample. The above probabilistic features are then concatenated with the 2048-dimensional spatial features to form the final combined feature vector per image with a dimension of 2050.

### 4.8 Feature Space Analysis of the Proposed Feature

**Fig. (7):**
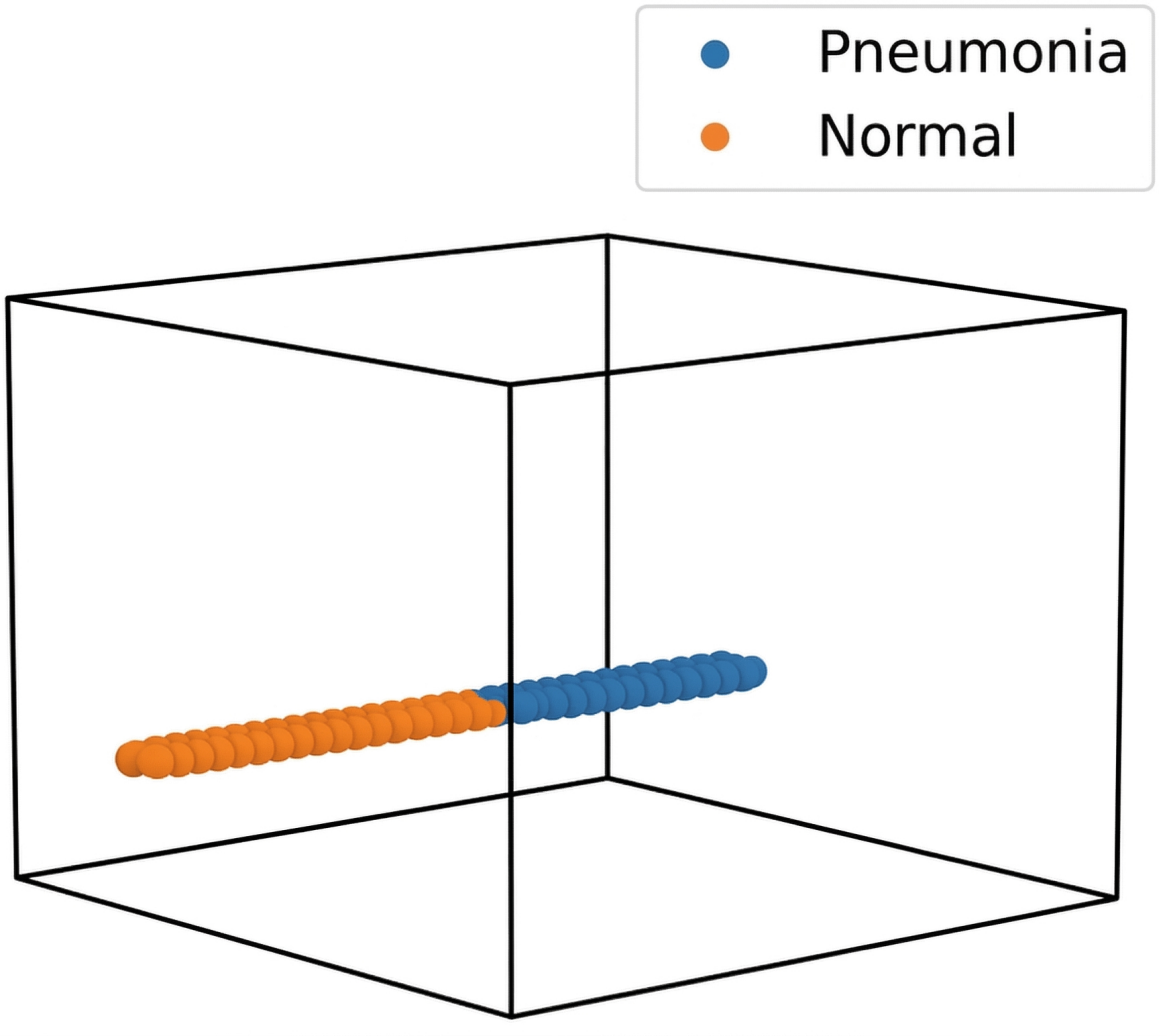
Feature space analysis extracted a new feature from X-ray scan images.

The provided feature space analysis is used to graphically display and analyze the spatial distribution of chest X-ray characteristics [30], offering a thorough examination of their spatial placement. The data representing chest X-ray features is intricately depicted by creating a 3D graph plot and using the feature space function to construct a comprehensive three-dimensional scatter plot. The careful use of two distinct marker types, clearly distinguished by color, highlights the distinctive properties of each data point, allowing for a clear distinction between individuals with pneumonia and those categorized as healthy. The examination specifies the ideal number of clusters necessary to differentiate between pneumonia-positive and healthy cases. This research methodology employs a distinctive spatial feature analysis to enhance the differentiation between individuals with and without pneumonia, using diagnostic chest X-ray imaging characteristics. Figure **7** illustrates an evaluation of the feature space for the newly created transfer feature set. The analysis demonstrates that the attributes obtained by the XceptRf-Net technique exhibited a significant level of distinct differentiation. The clear and separate categorization of these characteristics is crucial for enhancing the precision of diagnosing pneumonia in patients.

## 5 LIMITATIONS

The presented framework shows good performance on the chosen pediatric CXR dataset. The model’s generalization capability across multiple imaging sources, age ranges, or acquisition protocols (e.g., different resolutions, scanners, or clinical settings) has yet to be extensively investigated. This work is computationally driven and has not been directly related or validated with the medical practitioners. Given the promising performance statistics, however, the inclusion of radiologist feedback and future prospective clinical studies would be necessary for the implementation of the model for actual diagnostic approaches.

## CONCLUSION

This study presents a method for early identification of pneumonia using deep learning and machine learning. Our study tests included 5863 X-ray scans of children with pneumonia and healthy children aged 0 to 5. We introduce XceptRf-Net, a feature generation technique for extracting spatial properties from image data. The spatial features are then combined to provide an ensemble feature set for RF. We employed sophisticated machine learning and deep learning algorithms to evaluate the performance of a newly generated set of features. We constructed a pair of sophisticated deep learning models and a trio of machine learning models by utilizing X-ray image data and freshly devised features. The appropriate methodologies are verified by K-fold cross-validation. In addition, we evaluated the computational complexity of each technique. Thorough research has demonstrated that the logistic regression method outperforms existing studies with a precision rate of 0.98%. The efficacy of each technique is assessed through the utilization of fine-tuning and K-fold cross-validation. The methods’ computational complexity is also evaluated.

### FUTURE WORK

We plan to enhance our proposed method by exploring several avenues for improvement. One key area is the expansion of the dataset to include a more diverse set of images and patient demographics, which will help improve the generalizability of the model. We also aim to investigate the impact of different hyperparameter settings and tuning methods on model performance to further optimize the system. Additionally, we will explore integrating advanced deep learning techniques and feature extraction methods to potentially boost accuracy and robustness.

## Figures and Tables

**Table 1:** The literature analysis based on state-of-the-art approaches performance.

**Ref**	**Year**	**Proposed Technique**	**Dataset**	**Score**	**Limitations**
[20]	2021	Ensemble Model	Chest X-Ray dataset	94.84%	Computational cost is increased.
[21]	2021	Convolutional Neural Network	Chest X-Ray dataset	90.0%	Classical Methods are used.
[22]	2019	SVM+CNN	Early-stage chest images	80.56%	The Proposed Technique did not achieve a high score.
[14]	2024	EfficientNet-V2L	Chest X-Ray dataset	97.0%	Computational cost is increased.
[23]	2022	Federated Learning	Local hospital lab dataset	91.0%	The quantity of the dataset is low.
[12]	2023	RNN and LSTM	CXIP and C19Rd dataset	92.0%	Computational cost is increased.
[13]	2021	CNN+PCA	X-Ray Images dataset	96.57%	Low image quality decreases the performance.
[15]	2021	VGG+RF	Chest Radiographs dataset	95.4%	The generalizability of the dataset is low.
[17]	2022	CNN	CT and X-Ray dataset	97.0%	Computational cost is increased.
[18]	2021	CNN	CT images dataset	96.0%	Classical technique is used.
[19]	2024	Vision Trans- former	CXR dataset	97.0%	Computational cost is increased.

**Table 2:** This study shows the hyperparameter values of the applied models.

**Technique**	**Hyperparameter**
*KNC*	’n*_n_eighbour* = 5*,weights* =^′^ *uniform*^′^*,metric* =^′^ *minkoski*^′^*,leaf_s_ize* = 30*,p* =^′^ 2^′^
*LR*	copy*_x_* =^′^ *Truefitintercept* =^′^ *True*^′^*,Positive* =^′^ *False*^′^*,Normalize* =^′^ *False*^′^
*RF*	max depth=20,random*_s_tate* = 0*,nestimators* = 100*,criterion* =^′^ *gini*^′^*,maxfeatures*^′^*sqrt*^′^.
*MLP*	activation=’relu’,optimizer=’adam’, alpha=0.0001,learning rate=’constant’.
*Xception*	input shape=”256,256,3”, hidden layer sizes=(100), activation=’sigmoid’, Optimizer=’Adam’, alpha=0.0001, learning rate=’constant’.

**Table 3:** The experimental setup information.

**Specification**	**Value**
*Model*	Dell Intel(R) CPU 2.20Ghz
*ProgrammingLanguage*	Python 3.0
*CPUMHZ*	2200
*Cachevolume*	5632KB
*RAM*	13 GB
*CPUcore*	1
*Address Volume*	48bits Virtual, 48 bits Physical

**Table 4:** Shows xception models' accuracy, precision, recall, and F1 score results.

**Technique**	**Accuracy (%)**	**Precision (%)**	**Recall (%)**	**F1 (%)**
Xception	94	94	94	94

**Table 5:** Performance evaluation of machine learning models using spatial features.

**Models**	**Accuracy**	**Target Class**	**Precision**	**Recall**	**F1Score**
KNC	0.71	Pneumonia Normal Average	0.72 0.70 0.72	0.72 0.69 0.72	0.73 0.69 0.72
LR	0.72	Pneumonia Normal Average	0.71 0.68 0.72	0.72 0.69 0.72	0.71 0.69 0.72
RF	0.84	Pneumonia Normal Average	0.84 0.80 0.84	0.83 0.81 0.84	0.84 0.81 0.83
MLP	0.90	Pneumonia Normal Average	0.90 0.88 0.90	0.89 0.69 0.89	0.89 0.70 0.90

**Table 6:** Performance analysis using our proposed new transfer features.

**Models**	**Accuracy**	**Target Class**	**Precision**	**Recall**	**F1Score**
KNC	0.96	Pneumonia Normal Average	0.96 0.94 0.96	0.96 0.94 0.96	0.96 0.94 0.96
LR	0.98	Pneumonia Normal Average	0.98 0.98 0.98	0.98 0.97 0.98	0.98 0.97 0.98
RF	0.97	Pneumonia Normal Average	0.97 0.98 0.97	0.96 0.97 0.97	0.97 0.97 0.97
MLP	0.96	Pneumonia Normal Average	0.98 0.96 0.98	0.98 0.97 0.98	0.97 0.96 0.98

**Table 7:** K-fold cross-validation results for various techniques showing the stability and reliability of the proposed method.

**Technique**	**K-Fold**	**Accuracy (%)**	**Standard Deviation (%)**
*KNC*	2 3 5	94.5 95.3 95.8	±0.0112 ±0.0091 ±0.0073
-	10	96.0	±0.0062
*LR*	2 3 5	97.2 97.6 97.9	±0.0104 ±0.0082 ±0.0065
-	10	98.0	±0.0059
*RF*	2 3 5	96.0 96.5 96.8	±0.0121 ±0.0097 ±0.0083
-	10	97.0	±0.0078
*MLP*	2 3 5	97.0 97.5 97.8	±0.0110 ±0.0085 ±0.0071
-	10	98.0	±0.0061

**Table 8:** Our novel proposal shows computational complexity analysis.

**Technique**	**Computation Time**
*KNC*	0.0186
*LR*	0.01614
*RF*	1.3787
*MLP*	2.8900

**Table 9:** Proposed approach comparison with past applied state-of-the-art approaches.

**Ref**	**Proposed Technique**	**Dataset**	**Accuracy** **(%)**	**Precision** **(%)**	**Recall** **(%)**	**F1 Score** **(%)**
[20]	Ensemble Model	Chest X-Ray dataset	94.84	96	95	94
[21]	CNN	Chest X-Ray dataset	90	91	93	92
[22]	SVM + CNN	Early stage pneumonia images	80.56	90	91	92
[17]	CNN, PCA	CT and X-ray dataset	85	88	86	85
**[Our]**	**XceptRfNet+LR**	**Pediatric pneumonia X-Ray dataset**	**98**	**99**	**99**	**98**

## Data Availability

The dataset used in this study, Chest X-Ray Images (Pneumonia), is publicly available on Kaggle (https://www.kaggle.com/datasets/paultimothymooney/chest-xraypneumonia/data).

## LIST OF ABBREVIATIONS

AUC: Area Under the Curve
CNN: Convolutional Neural Network
CXR: Chest X-Ray
XceptRF-Net: Hybrid Approach Combining Xception and Random Forest Algorithms
RF: Random Forest
KNC: K-Nearest Neighbors Classifier
MLP: Multi-Layer Perceptron
LR: Logistic Regression
SVM: Support Vector Machine
ReLU: Rectified Linear Unit
FL: Federated Learning
ViT: Vision Transformer
F1 Score: Measure of Model’s Accuracy Balancing Precision and Recall
ROC: Receiver Operating Characteristic
Gini: Gini Impurity
PCA: Principal Component Analysis
SWE: Shear-Wave Elastography
